# Synergistic Enhancement of *Eimeria maxima* Vaccine Efficacy Through EF-1α Antigen and Chicken XCL1 Chemokine Adjuvant Combination

**DOI:** 10.3390/ani15162330

**Published:** 2025-08-08

**Authors:** Rong Chen, Xiao-Feng Lin, Hong-Yan Wu, Li-Na Li, Lei Wang, Deng-Feng Wang, Hai-Yan Wu, Pan-Pan Guo, Muhammad Mohsin, Guang-Wen Yin

**Affiliations:** 1 College of Animal Sciences, Fujian Agriculture and Forestry University, Fuzhou 350002, China; 18050199706@163.com (R.C.); 19959156561@163.com (X.-F.L.); abby8008@126.com (H.-Y.W.); 13625045210@163.com (L.-N.L.); dkwangl@fafu.edu.cn (L.W.); 000q820018@fafu.edu.cn (D.-F.W.); wuhaiyan_linda@fafu.edu.cn (H.-Y.W.); vetgpp@163.com (P.-P.G.); 2 Department of Parasitology, University of Agriculture, Faisalabad 38040, Pakistan

**Keywords:** *Eimeria*, chicken, subunit vaccine, adjuvant, immune response

## Abstract

Coccidiosis is a major parasitic disease that suppresses poultry productivity and causes significant global economic losses. Currently, controlling *Eimeria* parasites relies primarily on the use of anticoccidial drugs or live vaccines. However, these conventional control strategies face the dual constraints of escalating drug resistance and unsustainable economic expenditures. In this study, the efficacy of a chimeric subunit vaccine comprising *Eimeria maxima* Elongation Factor-1α (EmEF1α) and chicken chemokine Ligand-1 (ChXCL1) was assessed for protection against experimental *Eimeria maxima* infection. The synthetic gene fragment ChXCL1-EmEF1α was ligated to the pET28a vector and expressed in vitro. Western blot analysis confirmed the successful expression of the recombinant ChXCL1-EmEF1α protein. Chickens immunized with the ChXCL1-EmEF1α exhibited a significantly stronger IgY response and higher secretion of IL-2 and IL-17 compared to those vaccinated with recombinant ChXCL1 alone or challenged solely with *E. maxima*. Furthermore, the ChXCL1-EmEF1α group demonstrated enhanced anticoccidial effects, including reduced intestinal lesions, higher body weight gain, and lower oocyst shedding compared to control groups. Following *E. maxima* challenge, the EmEF1α and ChXCL1-EmEF1α groups demonstrated robust protective efficacy, achieving high ACI values of 182 and 178, respectively. In contrast, the ChXCL1 and UC groups exhibited significantly lower ACI values (150 and 149, respectively), indicating minimal protection. This improvement was also reflected in the immune response, with significantly elevated levels of CD4^+^ and CD8^+^ T cells in the ChXCL1-EmEF1α-treated chickens. Moreover, ChXCL1 acts as an effective adjuvant when fused with EmEF1α, enhancing the vaccine’s anticoccidial efficacy. These results suggest that the ChXCL1-EmEF1α chimeric immunogen is a promising candidate for developing subunit vaccines against *E. maxima* infections.

## Simple Summary

Chicken coccidiosis is caused by *Eimeria* parasites; this disease can lead to reduced productivity and economic losses. The current primary strategies for controlling *Eimeria* parasites involve administering anticoccidial drugs or live vaccines, and there is increasing demand for more scalable and cost-effective vaccines. In this study, we developed an innovative “mix-and-match” vaccine to combat *Eimeria maxima* infection. This fusion vaccine combines a parasite protein (*Eimeria maxima* Elongation Factor-1α, EmEF1α) that trains the immune system to recognize the pathogen with a chicken immunity booster (chicken chemokine ligand 1, ChXCL1) to enhance efficacy. Results show vaccinated chickens exhibited reduced gut damage, improved weight gain, and fewer parasites in their droppings, alongside increased production of disease-fighting cells (CD4^+^/CD8^+^ T cells) and antibodies. The combined approach outperforms EmEF1α alone, providing farmers with a more effective tool to safeguard poultry health.

## 1. Introduction

Chicken coccidiosis, caused by protozoan parasites of the genus *Eimeria*, specifically infects the intestinal tract of chickens [1,2,3]. These parasites invade intestinal epithelial cells, triggering inflammation, damaging the digestive tract, impairing feed conversion efficiency, and in severe cases, leading to mortality [4]. Clinical signs include enteritis and reduced weight gain, making coccidiosis a major threat to the poultry industry and a significant source of global economic losses [5]. Currently, seven *Eimeria* species (*E. tenella*, *E. acervulina*, *E. maxima*, *E. brunetti*, *E. mitis*, *E. necatrix*, and *E. praecox*) are known to infect chickens. Among these, *E. tenella*, and *E. necatrix* exhibit the strongest pathogenicity. *E. maxima* exhibits moderate pathogenicity, and possesses the highest immunogenicity. *E. maxima* primarily parasitizes the jejunum, the middle segment of the small intestine. This infection induces thickening and enlargement of the intestinal wall and is associated with the presence of yellowish-green mucoid exudate.

In the era of increasing anticoccidial drug resistance, vaccination has emerged as an attractive alternative approach for parasite control [6,7]. Live oocysts vaccination is the conventional approach to control chicken coccidiosis, but this method has safety risks and production constraints [8]. These challenges have intensified research into next-generation vaccine platforms, with subunit vaccines incorporating parasite-derived immunodominant antigens or recombinant DNA-expressed proteins emerging as particularly viable solutions, offering advantages in safety, manufacturing standardization, and potential cross-strain protection [9]. EF-1α, identified as the target of monoclonal antibody 6D-12-G10, exhibits broad reactivity against antigens within the apical complex across seven *Eimeria* species infecting chickens. In vitro, the chicken anti-EF-1α monoclonal antibody (6D-12-G10 mAb) effectively inhibits the invasion of *Eimeria acervulina* in chickens [10]. Immunoproteomic analysis by Liu et al. (2017) identified elongation factor-1α (EF-1α) as a conserved immunodominant antigen across *E. acervulina* and *E. maxima*; they also identified the same type of protein (EtEF-2) as a common immunodominant antigen in *E. tenella*, *E. acervulina*, and *E. maxima* [11]. Complementing this, Juarez-Estrada et al. (2023) mapped the immunodominant mimotopes from EtEF-2 via phage display screening [12]. Furthermore, administration of chicken with *Eimeria* EF-1α in combination with chIL-7 and cNK-2 peptides induces protective immunity against coccidial challenges [13]. Similarly, immunization with *Eimeria tenella* EF-1α recombinant protein induces protective immunity against *E. tenella* and *E. maxima* infections [14]. Collectively, these findings demonstrate that EF-1α elicits cross-protective immunity against avian coccidiosis, establishing its potential as a candidate vaccine antigen for controlling *Eimeria* infections.

XCR1 serves as the exclusive receptor for XCL1, functioning as a canonical transmembrane G-protein coupled receptor with high specificity [15]. It exhibits selectively conserved expression on cross-presenting dendritic cells (DCs) across multiple mammalian species including humans, mice, sheep, and macaques, where it mediates effective antigen presentation [16]. Current DC-targeting strategies primarily utilize viral vectors, receptor–ligand interactions, or C-type lectin receptors. The XCR1-XCL1 axis operates through receptor–ligand mediation: antigen conjugation to the ligand binds DC surface receptors, stimulating CD8^+^ T cells to induce immune responses. The XCL1-XCR1 axis efficiently mediates cytotoxic CD8^+^ T-cell responses. Intradermal injection of OVA with mXCL1-V21C/A59C adjuvant promotes accumulation and migration of CD103^+^XCR1^+^ DCs to draining lymph nodes, effectively inducing OVA-specific effector and memory CD8^+^ T cells [17]. XCL1-fused HA nucleic acid vaccines boost cytotoxic CD8^+^ T-cell proliferation, elevate IgG2a/IgG1 ratios, induce Th1-biased CD4^+^ responses with increased IFN-γ secretion, and confer protection against lethal influenza challenge [18]. Avian XCL1-mediated DC-targeting vaccines remain unreported. One existing application includes the co-administration of pcDNA3.1-*Eimeria* vaccine with XCL1 in chickens, which reduces oocyst shedding and improves weight gain post-*Eimeria acervulina* infection [19]. FLT3-high chicken cells exclusively express XCR1, confirming conserved surface phenotypes between avian cDCs and mammalian XCR1^+^ cDC1 subsets [20]. This supports XCL1′s utility in poultry immunology.

The immune response to coccidiosis in chickens exhibits interspecies and stage-specific characteristics, with only a limited degree of cross-immunity. The protective immunity triggered by coccidia is generally mediated through both cellular and humoral immune responses, though the exact mechanisms remain unclear. The prevailing view holds that cellular immunity plays a dominant role in conferring protection against coccidiosis [21]. Subunit vaccines offer a biologically safe and economically viable control option for coccidiosis, enabling researchers to select from the most effective antigens for this pathogen. However, they tend to be poorly immunogenic and require the incorporation of adjuvants into the vaccine formulation [22]. Recent advances have focused on identifying novel adjuvants that enhance both humoral and cell-mediated immune responses to improve vaccine efficacy [23,24]. We hypothesized that chicken XCL1 (ChXCL1) could amplify the immunogenicity of *Eimeria maxima* EF-1α (EmEF1α), thereby inducing a protective immune response in chickens. To test this hypothesis, we examined the adjuvant potential of ChXCL1 when combined with EmEF1α and evaluated the protective effects of the recombinant ChXCL1-EmEF1α fusion protein in chickens.

## 2. Materials and Methods

### 2.1. Animals and Parasites

One-day-old, male, Hy-Line brown layer chickens were purchased from Fuzhou Poultry Breeding company (Fuzhou, China). They were reared in a coccidia-free room and had free access to food and water without anticoccidial drugs during the experiment. Prior to the experiment, fecal samples from the chicks were examined to confirm the absence of *E. maxima* infection.

The *E. maxima* oocysts (Beijing strain) were provided by Xun Suo’s laboratory at China Agricultural University. To ensure oocyst viability before the experiment, *E. maxima* oocysts were given to 14-day old chicks orally (10^4^ oocysts per bird). At 6 to 9 days post-infection, the unsporulated oocysts were purified from the fecal samples through saturated sodium chloride flotation, following established protocols to facilitate sporulation [25]. Then, the *E. maxima* sporulated oocysts were identified by microscopical and molecular methods. Only oocysts with a sporulation rate exceeding 85% were selected and used in this study.

### 2.2. Plasmid Construction

The ChXCL1-EmEF1α (comprising the removal of a signal peptide of ChXCL1) fragment was synthesized in vitro by Sangon Biotech (Shanghai, China) Co., Ltd. according to the gene sequences of ChXCL1 (NP_990377) and EmEF1α (XP_013336300.1) in NCBI database. After double digestion with Nde I and Xho I restriction enzymes, the synthesized fragment was ligated to pET28a empty vector by T4 ligase to create pET28a-ChXCL1-EmEF1α. Through amplification, the target fragment (ChXCL1 and EmEF1α) was connected to the prokaryotic expression vector pET28a to construct recombinant plasmids pET28a-ChXCL1 and pET28a-EmEF1α. Primers were designed based on the EmEF1α and ChXCL1 gene sequences, incorporating Nde I and Xho I restriction sites. The primer sets with features for analyzing Tm, GC content, secondary structures, and specificity were designed by Primer Premier 5 software. The open reading frames (ORFs) of EmEF1α and ChXCL1 were amplified via PCR, with primer sequences listed in Table 1. PCR products were electrophoresed on 1% agarose gels, and target bands were excised, purified, and cloned into the pEASY-Blunt Simple Cloning Vector (TransGen Biotech, Beijing, China) for sequencing.

### 2.3. Expression and Purification of Recombinant Proteins

The EmEF1α and ChXCL1 genes were inserted into the Nde I/Xho I-digested pET28a vector, generating pET28a-EmEF1α and pET28a-ChXCL1. These constructs (pET28a-ChXCL1-EmEF1α, pET28a-EmEF1α, and pET28a-ChXCL1) were transformed into *E. coli* BL21 (DE3) for protein expression. Bacterial cultures were grown to mid-log phase, induced with 1.0 mM IPTG for 6 h at 37 °C, and harvested by centrifugation. Cells were lysed by sonication on ice, and recombinant His_6_-tagged proteins were purified from the soluble fraction using a Hi-Trap metal-chelating column (TransGen Biotech, Beijing, China) [25]. The concentrations of eluted proteins were estimated using the Bradford assay method (Bradford assay kit, Amresco, Boise, ID, USA). Protein identities were verified by Western blotting.

### 2.4. Vaccination and Challenge Infection

Five groups (fifteen chickens with a similar weight range per group) of 14-day-old chickens were immunized intramuscularly in the breast as follows: Group 1: 200 μg EmEF1α emulsified in Freund’s complete adjuvant (FCA). Group 2: 200 μg ChXCL1-EmEF1α (without adjuvant). Group 3: unimmunized–challenged (UC) group. Group 4: unimmunized–unchallenged (UU) group. Group 5 (Adjuvant control): 200 μg ChXCL1 alone. Fourteen days post-primary immunization, birds were boosted with the same dose. Blood samples were collected from the wing vein of six birds from each group for CD4^+^/CD8^+^ T-cell subset analysis via flow cytometry. At 14 days post-boost, all groups (except the unchallenged control) were orally challenged with 5 × 10^4^ sporulated *E. maxima* oocysts. Feed and water were withdrawn on the evening of day 5 post-challenge. On day 6, five birds per group were randomly selected and euthanized by cervical dislocation for intestinal lesion assessment and H&E staining. The abdominal cavity was opened by incision along the sternal tip. Jejunum lesions were scored (0–4 scale) by three blinded examiners [26]. A 2 cm intestinal segment anterior to the vitelline diverticulum was excised and fixed in neutral buffered 10% formalin solution at room temperature for H & E staining (Fuzhou Dubite Biotechnology Co., Ltd., Fuzhou, China). Body weight was individually recorded at 0 (before *E. maxima* challenge) and 10 d post-*E. maxima* challenge, and then the average body weight per chicken was calculated. Average weight gain (AWG) was calculated as follows: (final total body weight per group—initial total body weight per group)/number of chickens per group. Rate of relative weight gain (RWG) was calculated as follows: (AWG of immunized or infected group/AWG of negative control group) × 100%. Feces from each group were collected separately at days 6–8 post-challenge. Oocyst shedding per bird was determined using a McMaster chamber. The oocysts reduction ratio was calculated according to the following formula: [(mean oocyst count of the challenged control group—mean oocyst count of the vaccinated group)/mean oocyst count of the challenged control group] × 100%. The anticoccidial index (ACI) was used to evaluate the anticoccidial efficacy. The ACI is calculated as follows: ACI = (survival rate + RWG) × 100—(lesion value + oocyst value) [27]. The anticoccidial effect of chickens was considered excellent when ACI ≥ 180, good when 160 ≤ ACI < 180, fair when 120 ≤ ACI < 160, and no anticoccidial effect when ACI < 120 [28].

### 2.5. Flow Cytometric Analysis of CD4^+^ and CD8^+^ T Cell Populations

Peripheral blood mononuclear cells (PBMCs) were isolated from chicken whole blood two weeks post-secondary immunization using a lymphocyte separation kit (Solarbio, Beijing, China). The isolated cells were washed and resuspended in PBS, then stained with fluorochrome-conjugated antibodies (PE-CD3, APC-CD4, and FITC-CD8a) (Southern Biotechnology Associates, Birmingham, AL, USA) for 30 min at room temperature in the dark [29]. Cell populations were analyzed using NovoCyte Flow Cytometer (ACEA Biosciences, Inc., San Diego, CA, USA) with NovoExpress^®^ software (version 1.5.0).

### 2.6. Serum Anti-EF1α Antibody Detection

Anti-EF1α IgY antibody titers were determined by indirect ELISA [25]. Briefly, 96-well microplates were coated with 2.0 μg/well of purified EF1α protein in carbonate–bicarbonate buffer (pH 9.6) overnight at 4 °C. After washing with PBST (PBS containing 0.05% Tween-20), plates were blocked with 200 µL of PBST containing 5% bovine serum albumin (BSA) (Solarbio, Beijing, China) for 2 h at 37 °C. Serum samples (1:100 dilution in PBS) from immunized chickens were added (100 μL/well) and incubated for 1 h at 37 °C. Following five PBST washes, HRP-conjugated goat anti-chicken IgY (1:10,000; TransGen Biotech, Beijing, China) was added and incubated for 1 h at 37 °C. Non-infected chicken serum (1:100 dilution) and PBS were used as controls during the analysis. The reaction was developed using standard TMB substrate and stopped with 2M H_2_SO_4_. Absorbance was measured at 450 nm using a microplate reader (BioTek Instruments, Charlotte, VT, USA).

### 2.7. Serum Cytokine Profiling

Concentrations of chicken IL-2, IL-4, IL-10, IL-17, and IL-12 in serum samples were quantified using commercial ELISA kits (CusaBio, Wuhan, China) according to the manufacturer’s protocols. Firstly, standard wells and test sample wells were set up, then 50 µL of test sample or serially diluted standards were added into the wells, respectively; following this, 50 µL enzyme conjugate and 50 µL detection antibody were added into each well and these wells were incubated for 1 h at 37 °C. After washing, 50 µL of chromogen A solution and 50 µL of chromogen B solution were added into each well, gently mixed and incubated for 15 min at 37 °C. Finally, 50 µL of the stop solution was added into each well and the OD values of each well were determined using a microtiter plate reader at 450 nm. The comparative analysis and bar plot were produced using the ggpubr function of R (Rversion R-3.4.3). All samples were analyzed in duplicate, with cytokine concentrations calculated from standard curves.

### 2.8. Statistical Analysis

Data are presented as mean ± standard deviation (SD). Statistical significance was determined by one-way ANOVA followed by Tukey’s post hoc test using SPSS software (version 25.0, IBM, Armonk, NY, USA). Graphical representations were generated using GraphPad Prism 8 (GraphPad Software). Differences were considered statistically significant at *p* < 0.05.

## 3. Results

### 3.1. Cloning of ChXCL1-EmEF1α, ChXCL1, EmEF1α, and Recombinant Plasmids Construction

**Figure 1:**
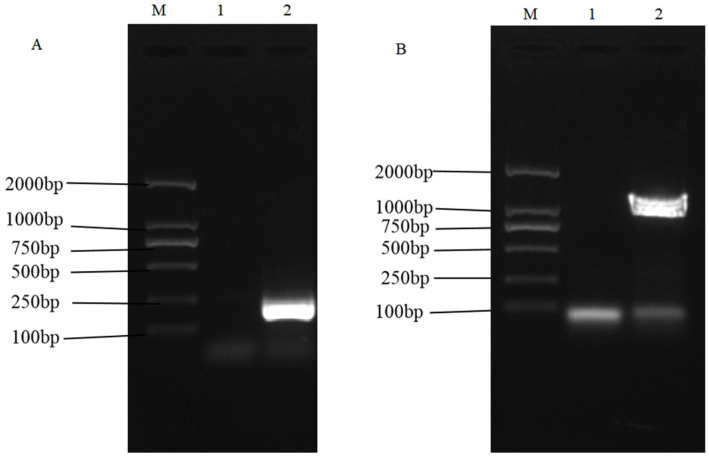
ChXCL1 and EmEF1α gene amplification and gel electrophoresis. (**A**) Amplification of ChXCL1 gene. M: Trans2K DNAMark. Lane 1: Negative control; Lane 2: Amplification product of ChXCL1 (231 bp). (**B**) Amplification of EmEF1α gene. M: Trans2K DNAMark. Lane 1: Negative control; Lane 2: Amplification product of EmEF1α (1362 bp).

The ChXCL1-EmEF1α fragment was synthesized in vitro. ChXCL1 and EmEF1α were successfully amplified using the synthesized gene of ChXCL1-EmEF1α. The result of the agarose gel electrophoresis showed that ChXCL1 had a size of 231 bp (Figure 1A) and EmEF1α had a size of 1362 bp (Figure 1B), which corresponds to the molecular weight of each gene. Subsequently, the ChXCL1-EmEF1α, ChXCL1, and EmEF1α gene fragments were ligated with the pET-28a plasmid, respectively. The success of the recombinant plasmids of pET-28a-ChXCL1-EmEF1α, pET-28a-ChXCL1, and pET-28a-ChXCL1 were confirmed by restriction enzyme digestion and sequence analysis.

### 3.2. Identification of Recombinant Proteins

**Figure 2:**
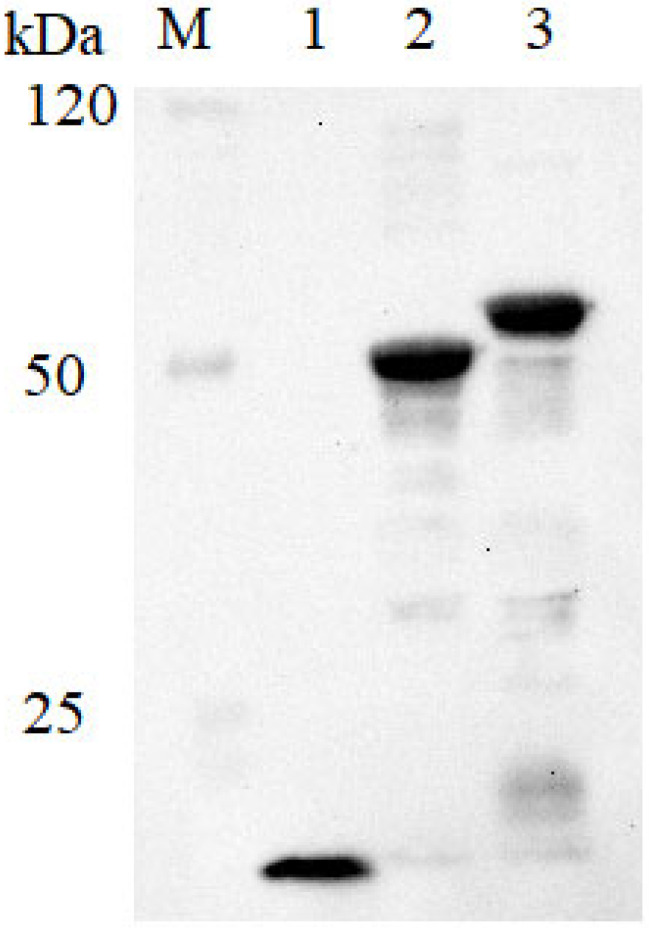
Protein expression in Western blot. M = marker, pET28a-XCL1 (line 1), pET28a-EmEF1α (line 2), and pET28a-ChXCL1-EmEF1α (line 3) were established by the Western blotting method.

The recombinant proteins ChXCL1, EmEF1α, and ChXCL1-EmEF1α were successfully expressed as His_6_-tagged fusion proteins in *E. coli* BL21(DE3) and purified using nickel affinity chromatography. Western blot analysis using anti-His_6_ monoclonal antibody verified the identity of all three recombinant proteins with molecular weights of approximately 8 kDa (ChXCL1), 50 kDa (EmEF1α), and 58 kDa (ChXCL1-EmEF1α) (Figure 2).

### 3.3. ChXCL1-EmEF1α Vaccine Efficacy Against E. Maxima in Chicken

Evaluation of vaccine efficacy revealed significant protection against *E. maxima* infection in immunized chickens. Birds vaccinated with either EmEF1α or ChXCL1-EmEF1α fusion protein exhibited markedly improved outcomes compared to control groups, including the following: (1) significantly higher body weight gain (*p* < 0.05), (2) reduced intestinal lesion scores (*p* < 0.05), and (3) decreased oocyst shedding with reduction rates ranging from 0.83% to 61.33% (Table 2). The anticoccidial index (ACI) is a key indicator for evaluating anticoccidial vaccine efficacy. Following *E. maxima* challenge, the EmEF1α and ChXCL1-EmEF1α groups achieved high ACI values of 182 and 178, respectively, demonstrating strong protective efficacy. In contrast, the ChXCL1 and UC groups showed significantly lower ACI values (150 and 149, respectively), indicating minimal protective efficacy (Table 2). Notably, the protective efficacy of ChXCL1-EmEF1α was comparable to that of EmEF1α emulsified in Freund’s complete adjuvant.

### 3.4. Evaluation of Intestinal Lesions

**Figure 3:**
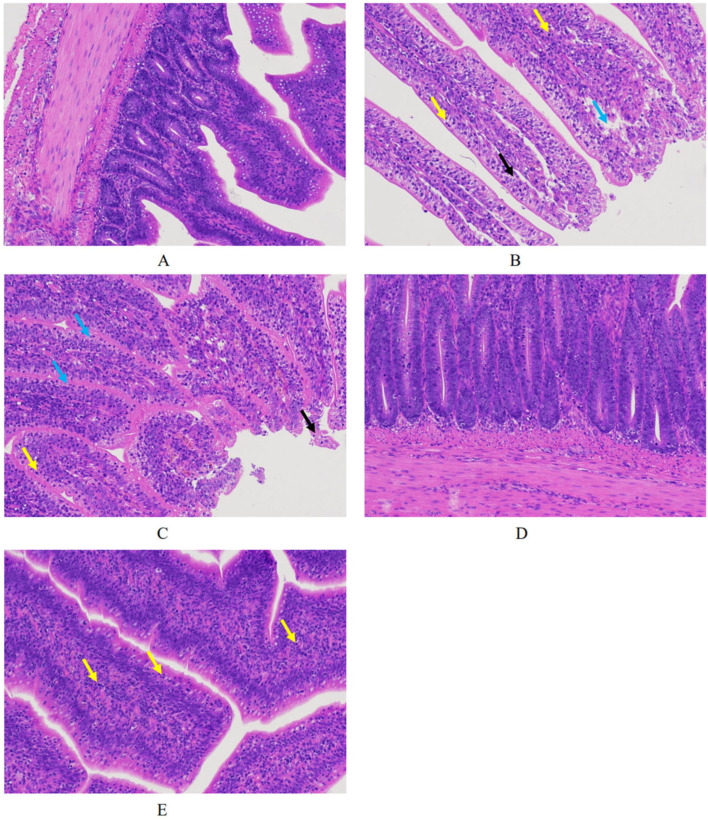
Histopathological examination of intestinal tissues in various experimental groups. (**A**) UU group; (**B**) UC group; (**C**) ChXCL1 group; (**D**) EmEF1α group; (**E**) ChXCL1-EmEF1α group. Yellow arrows represent lamina propria lymphocyte infiltration; blue arrows represent mucosal epithelial detachment; black arrows represent epithelial cell shedding with cytoplasmic alterations.

Histopathological examination of intestinal tissues provided further evidence of vaccine protection (Figure 3). While unchallenged controls showed normal intestinal architecture with intact villi (Figure 3A), challenged controls displayed pathological changes including mucosal epithelial detachment (Figure 3B, blue arrow), epithelial cell shedding with cytoplasmic alterations (Figure 3B, black arrow), and lamina propria lymphocyte infiltration (Figure 3B, yellow arrow). The ChXCL1 group exhibited similar lesions as that in infected controls (Figure 3C). In contrast, chickens immunized with EmEF1α (Figure 3D) or ChXCL1-EmEF1α (Figure 3E) showed only minimal histological changes, limited to slight intraepithelial lymphocyte infiltration.

### 3.5. CD4^+^ and CD8^+^ T Cell Profile Analysis Using Flow Cytometry

**Figure 4:**
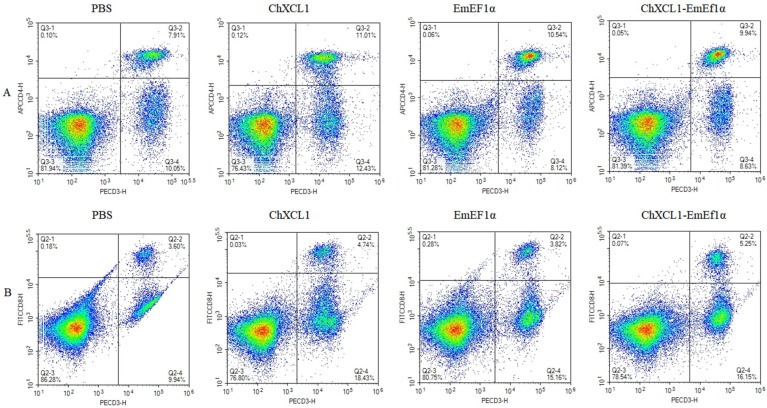
The proportion of the T cell subpopulation in chickens immunized with PBS, ChXCL1, EmEF1α, and ChXCL1-EmEF1α was determined by flow cytometry 14 days after the second immunization. (**A**) Detection of CD3 ^+^ CD4^+^T lymphocytes in immunized chickens. (**B**) Detection of CD3 ^+^ CD8^+^T lymphocytes in immunized chickens.

Flow cytometry was used to evaluate the proportion of CD4^+^ and CD8^+^ T lymphocytes in immunized chickens. The results are shown in Figure 4 and Table 3. All treatment groups showed markedly increased CD4^+^ T cell proportions compared to PBS controls (*p* < 0.05) (Table 3). The ChXCL1 and ChXCL1-EmEF1α groups demonstrated particularly robust CD8^+^ T cell responses, with statistically significant elevations relative to both PBS controls and the EmEF1α group (*p* < 0.05) (Table 3). The ChXCL1-EmEF1α fusion protein induced the most pronounced immunomodulatory effects, stimulating simultaneous upregulation of both CD4^+^ and CD8^+^ T lymphocyte subsets.

### 3.6. Quantification of Serum Anti-EF1α Antibodies by ELISA

**Figure 5:**
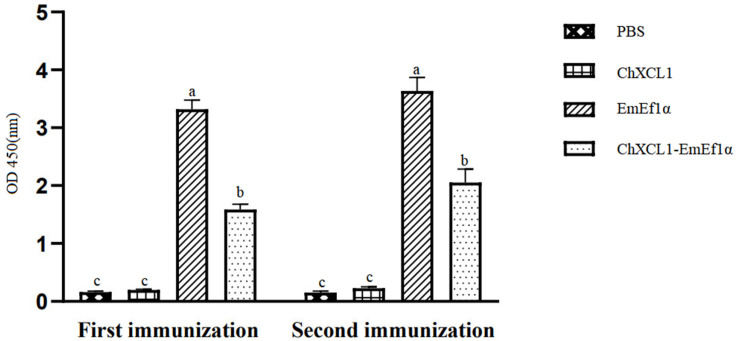
The IgY antibody level in chickens sera after vaccination. Significant difference (*p* < 0.05) between data was annotated with different letters. No significant difference (*p* > 0.05) between data was annotated with the same letter.

Serological analysis demonstrated strong humoral immune responses to vaccination. The EmEF1α group generated significantly higher anti-EF1α IgY titers than other groups at 14 days post-primary immunization (*p* < 0.05), with this difference persisting following booster immunization. The EmEF1α group consistently showed the highest antibody levels throughout the study period. Notably, there was no significant difference in IgY antibody levels between the PBS and ChXCL1 control groups (*p* > 0.05) (Figure 5).

### 3.7. Serum Cytokine Profile Analysis in Chickens Immunized with ChXCL1-EmEF1α Fusion Protein

**Figure 6:**
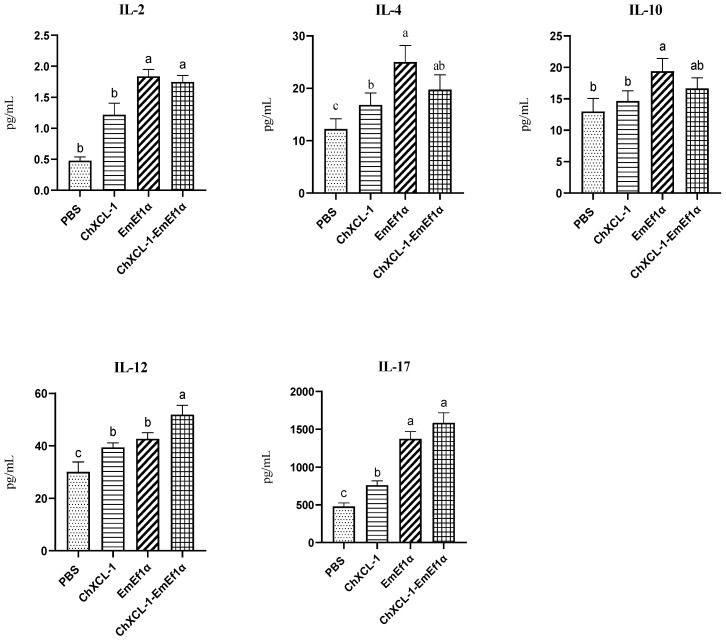
The concentration of cytokines IL-2, IL-4, IL-10, IL-12, and IL-17 in serum after the second immunization. Significant difference (*p* < 0.05) between data was annotated with different letters. No significant difference (*p* > 0.05) between data was annotated with the same letter.

The IL-2 level in EmEF1α group was the highest, followed by that in ChXCL1-EmEF1α group, with no significant difference between the two groups. The IL-2 levels in EmEF1α group and ChXCL1-EmEF1α group showed significant differences compared to the PBS group and ChXCL1 group (*p* < 0.05). The EmEF1α group exhibited the highest IL-4 levels, showing significant differences compared to the PBS control group and ChXCL1 adjuvant group (*p* < 0.05). The EmEF1α group showed maximal IL-10 secretion, followed closely by ChXCL1-EmEF1α. Both ChXCL1-EmEF1α and EmEF1α vaccines elicited robust Th1-type responses, as evidenced by significantly elevated IL-12 levels compared to controls (*p* < 0.05), with peak IL-12 production in the ChXCL1-EmEF1αgroup. While IL-17 levels remained stable in PBS and ChXCL1 groups, significant fluctuations occurred in vaccinated groups (*p* < 0.05) (Figure 6).

## 4. Discussion

Coccidiosis remains a major economic burden in intensive poultry production, primarily through reduced weight gain and intestinal lesions [30]. EF-1α was shown as a promising vaccine target approach against parasite species [13]. The conserved role of EF-1α in parasite development and host cell invasion makes it particularly suitable for vaccine development against diseases [14].

The observed immune responses in this study highlight the potential of XCL1 as a molecular adjuvant for enhancing vaccine efficacy against avian coccidiosis. The robust CD8^+^ T cell activation in ChXCL1-EmEF1α-vaccinated chickens underscores the ability of XCL1 to promote MHC-I cross-presentation, a critical mechanism for combating intracellular pathogens [17]. While both adjuvanted and non-adjuvanted formulations stimulated CD4^+^ T cell proliferation, the differential CD8^+^ T cell responses suggest that XCL1 uniquely enhances cytotoxic T lymphocyte (CTL) activity, which is essential for parasite clearance.

The cytokine profiles further validate the adjuvant-dependent divergence of immune responses. The pronounced Th1 bias prompted by ChXCL1-EmEF1α, evidenced by significantly elevated IL-12 levels, proves particularly beneficial for combating *Eimeria* infections, given that Th1-mediated cellular immunity plays a pivotal role in restricting parasitic proliferation [31]. Conversely, the FCA-adjuvanted EmEF1α formulation induced a mixed Th2/regulatory response, characterized by IL-4 and IL-10 production, which may enhance antibody generation. These finding suggest that XCL1 can not only amplify immunogenicity but can also direct the host’s immune response toward a more efficacious mechanism for eliminating pathogens [32].

Despite the superior Th1 response in the XCL1-adjuvanted group, the FCA formulation induced higher IgY titers, indicating that different adjuvants may be optimal depending on the desired immune outcome. However, the significant reduction in oocyst shedding (61.33%) and the high anticoccidial index (ACI = 178) in the ChXCL1-EmEF1α group demonstrate that cell-mediated immunity plays a dominant role in protection against *E. maxima* [33]. These findings are consistent with earlier studies showing that while antibodies contribute to immunity, T cell responses are more critical for resolving coccidial infections [34].

The conservation of XCL1 functionality in chickens expands the potential applications of this targeting strategy in avian vaccinology [20]. Given the success of XCL1 in enhancing both cellular and humoral immunity, future studies could explore its use in multivalent vaccines targeting multiple *Eimeria* species or other poultry pathogens. Additionally, optimizing delivery systems such as recombinant viral vectors or nanoparticle formulations could further enhance the stability and immunogenicity of XCL1-adjuvanted vaccines [35,36].

## 5. Conclusions

In this study, ChXCL1-EmEF1αand EmEF1αwere successfully expressed in *E. coli* and purified by Ni-NTA affinity chromatography. Vaccination trials demonstrated that ChXCL1-EmEF1α and EmEF1α conferred partial protection against *E. maxima* infection. Both cellular and humoral immunity was observed after immunization with ChXCL1-EmEF1α and EmEF1α. The specific IgY levels were significantly increased, and the proportions of CD8^+^ T cells and ACI in ChXCL1-EmEF1α-immunized chickens were effectively increased. These findings provide a foundation for developing future vaccines against coccidiosis and highlight the potential of XCL1 as a molecular adjuvant in veterinary vaccinology.

## Figures and Tables

**Table 1:** Primers used in this study.

Primer Name	Primer Sequence 5′ → 3′	Gene Bank Number
ChXCL1-*Nde I* -F	CATATGGTGGCAAGCCAG AGTATGCG	NP_990377
ChXCL1-*Xho I* -R	CTCGAGTTAACGACGGCG GGTGGT	
EmEF1α-*Nde I* -F	CATATGGGTAAAGAAAAA ACCCAT	XP_013336300.1
EmEF1α-*Xho I* -R	CTCGAGTTATTTTTTAGCT GCGGC	

**Table 2:** Protective effects of ChXCL1, EmEF1α, and ChXCL1-EmEF1α against experimental infection of *E. maxima* in chickens.

Groups	Average Amount of Oocyst (×10^7^) (*n* = 10)	Oocyst Reduction Rate (%)	Mean Lesion Score (*n* = 5)	Average Weight Gain (AWG) (*n* = 10)	Rate of Relative Weight Gain (RWG, %)	Anticoccidial Index (ACI)
EmEF1α	0.70	47.12	1.17 ± 0.07 ^b^	124.83 ± 10.74 ^c^	83.51	182
ChXCL1- EmEF1α	0.51	61.33	1.37 ± 0.67 ^b^	119.95 ± 5.82 ^c^	80.24	178
ChXCL1	1.31	0.83	2.87 ± 0.29 ^a^	80.65 ± 3.05 ^b^	53.95	150
UC	1.33	0	3.17 ± 0.18 ^a^	73.60 ± 1.48 ^b^	49.24	149
UU	0	100	0	149.48 ± 7.03 ^a^	100	-

Note: In every column, there was a significant difference (*p* < 0.05) between numbers with different letters. No significant difference was shown between values with the same letter.

**Table 3:** Lymphocytes profile in each groups.

Group	CD4^+^ (%)	CD8^+^ (%)	CD4^+^/CD8^+^
PBS	7.04 ± 0.86 ^a^	3.68 ± 0.73 ^a^	1.91
ChXCL1 group	10.85 ± 0.77 ^b^	5.00 ± 0.51 ^b^	2.17
EmEF1α group	11.53 ± 0.84 ^b^	3.88 ± 0.08 ^a^	2.97
ChXCL1-EmEF1α group	10.09 ± 0.73 ^b^	5.47 ± 0.30 ^b^	1.84

Note: Values are expressed as mean ± SD (*n* = 6). Means in the same column with different letters were significantly different between treatment groups (*p* < 0.05).

## Data Availability

The original contributions presented in this study are included in the article. Further inquiries can be addressed to the corresponding author.
